# Comment on: Comorbidities (excluding lymphoma) in Sjögren’s syndrome. Reply

**DOI:** 10.1093/rheumatology/keab603

**Published:** 2021-07-24

**Authors:** José M Pego-Reigosa, Íñigo Rúa-Figueroa Fernández de Larrinoa

**Affiliations:** Rheumatology Department, University Hospital Complex Vigo; IRIDIS (Investigation in Rheumatology and Immune-Mediated Diseases)-VIGO Group, Galicia Sur Health Research Institute (IISGS), Vigo, Spain; Rheumatology Department, Doctor Negrín University Hospital of Gran Canaria, Gran Canaria, Spain

Dear Editor, We read with interest the comment of Manzo *et al.* [1] on our review article ‘Comorbidities (excluding lymphoma) in Sjögren’s syndrome’ [2]. The authors highlight the importance of cognitive impairment (CI) as well as the different mechanisms leading to it, in patients with primary SS (pSS). We fully agree with them that neurological manifestations are frequently underestimated in these patients and that rheumatologists should pay special attention to their diagnosis.

The prevalence of neurological symptoms in pSS patients ranges between 8.5 and 70% [3]. The reasons for this wide range in the literature are the heterogeneity of the pSS classification criteria and of the definitions of the different neurological syndromes, and the diverse diagnostics tools used to assess those neurological symptoms in the different studies. In any case, about 20% of pSS patients may present clinically significant neurological involvement, which may be the first manifestation of the disease in >25% of the cases [4].

We also agree with the comment by Manzo *et al.* about the need for both a better assessment of cognitive dysfunction in pSS patients and searching for pSS in patients with CI, as they established in their systematic review [5].

Whether CI or other neurological manifestations should be considered ‘comorbidities’ defined as ‘simultaneous presence of more than one disease in a patient’ or only clinical features of pSS in a specific system could be discussed. However, we think that that debate should not minimize the relevance of the message of Manzo *et al.*, which highlights the great importance of neurological manifestations, and CI in particular, in pSS patients.

*Funding*: No specific funding was received to carry out the work described in this article.

*Disclosure statement*: The authors have declared no conflict of interest.

## Data availability statement

Data are available upon reasonable request by any qualified researchers who engage in rigorous, independent scientific research, and will be provided following review and approval of a research proposal and Statistical Analysis Plan (SAP) and execution of a Data Sharing Agreement (DSA). All data relevant to the study are included in the article.
